# The Effect of Anti-Rosetting Agents against Malaria Parasites under Physiological Flow Conditions

**DOI:** 10.1371/journal.pone.0073999

**Published:** 2013-09-16

**Authors:** Yvonne Adams, J. Alexandra Rowe

**Affiliations:** Institute of Immunology and Infection Research, Centre for Immunity, Infection and Evolution, School of Biological Sciences, University of Edinburgh, Edinburgh, United Kingdom; Liverpool School of Tropical Medicine, United Kingdom

## Abstract

Rosetting remains the dominant malaria parasite adhesion phenotype associated with severe disease and pathogenicity in Africa. The formation of rosettes, whereby a *Plasmodium falciparum* infected erythrocyte (IE) adheres to two or more non-IEs, is thought to facilitate the occlusion of microvascular blood vessels by adhering to host endothelial cells and other bound IEs. Current methods of determining the rosette-disrupting capabilities of antibodies/drugs have focused on static assays. As IEs *in vivo* are exposed to shear stresses within the microvasculature, the effect of flow conditions on rosetting requires further examination. This study establishes a new rosetting flow assay using a closed perfusion system together with inverted fluorescence microscopy and image analysis, and confirms previous reports that rosettes exist under shear stresses equivalent to those present in the microvasculature (0.5–1.0 dyn/cm^2^). Furthermore, we tested the effectiveness of rosette-disrupting PfEMP1 antibodies, heparin and fucoidan over a range of concentrations on two *P. falciparum* strains, and found no statistically significant differences between the results of static and flow assays. The new flow assay is a valuable addition to the tools available to study rosetting. However, the static assay has good predictive value and remains useful as the standard screening test for rosette-disrupting interventions.

## Introduction

*Plasmodium falciparum* is the most pathogenic of the *Plasmodium* species causing human malaria. Parasite survival in the human host is enhanced by its ability to adhere to various receptors expressed on endothelial cells, leading to sequestration of infected erythrocytes (IEs) in the microvasculature, allowing the parasite to avoid splenic clearance [1]. However, the presence of sequestered IEs in the microvasculature obstructs blood flow and can lead to hypoxia and metabolic acidosis, contributing to the patho-physiology of life-threatening malaria [2], [3]. Obstruction of small blood vessels by sequestrated IEs is further enhanced by the formation of rosettes [4], a mechanism whereby one IE binds two or more non-IE, leading to larger aggregates of cells, further compounding the risk of ischemic damage [5]. As a result, rosetting is the dominant parasite adhesion phenotype associated with severe malaria and pathogenicity in Africa [6], [7], [8]. Human erythrocyte polymorphisms that reduce the ability of *P. falciparum* to form rosettes, such as blood group O [9] and Complement Receptor One deficiency [10], confer significant protection against life-threatening malaria [10], [11].

Given the ability of rosetting IEs to occlude microvessels and restrict blood flow, the search for anti-rosetting agents to reverse adhesion and unblock the microvasculature could lead to new adjunctive treatments for severe malaria [12]. *In vitro* studies have identified sulphated glycoconjugate compounds such as heparin, fucoidan and curdlan sulphate as being effective at disrupting rosettes [13], [14], [15], [16]. Some of these sulphated polysaccharides not only disrupt rosetting, but also inhibit adhesion of IEs to host endothelial and placental receptors such as CSA and CD36 [17], [18], [19], [20]. However, these compounds suffer from disadvantages making them unsuitable for widespread clinical use, such as limited strain-specific activity (eg heparin and heparin-derivatives) [13], or anti-coagulant side effects (eg. curdlan sulfate) [16]. Therefore, although these compounds may have some potential as adjunctive therapies, the search for safe, effective, broad-spectrum anti-rosetting agents continues.

Recent advances in understanding the molecular mechanisms of rosetting may help in the rational development of new therapies. Rosetting is mediated by parasite molecules on the surface of IEs binding to receptors on non-IEs. The parasite adhesion molecules are specific members of the *Plasmodium falciparum* erythrocyte membrane protein 1 (PfEMP1) family, the major erythrocyte surface antigen expressed by the parasite [10]. PfEMP1 variants are high molecular weight (200–350 kDa) proteins containing multiple cysteine-rich extra-cellular domains, termed Duffy-Binding Like (DBL) domains [21]. Recent investigations have identified the N-terminal “NTS-DBLα” region of rosette-mediating PfEMP1 variants as the erythrocyte binding domain [10], [22], [23], [24]. Polyclonal antibodies generated against NTS-DBLα are able to inhibit rosette formation, and also disrupt pre-existing rosettes [25], [26]. More detailed work to localize binding sites within NTS-DBLα may lead to the development of novel rosette-disrupting therapeutics [27], [28].

Current *in vitro* assays for assessing the effect of anti-rosetting agents are carried out under static conditions. Drugs or antibodies are added to the rosetting parasite culture suspension in eppendorf tubes or 96 well plates, and after a short incubation, a wet preparation is made and viewed by microscopy. The percentage of mature IEs forming rosettes is counted to give the rosette frequency in the presence or absence of drug/antibody. *In vivo*, the environment is vastly different. The IEs are subjected to continuous flow of the vascular system, with shear stresses ranging from 0.5–5.0 dyn/cm^2^ within the microvasculature, to >10 dyn/cm^2^ on the arterial side of the circulation [29]. The rheological properties of rosettes have been examined previously using a variety of techniques including perfusion of *ex vivo* rat microvasculature [5], micropipette manipulation and use of a cone-plate viscometer [30], [31] and use of a Laser-Assisted Optical Rotational Cell Analyser (LORCA) [32]. Using these techniques it has been shown that rosettes can withstand shear forces equivalent to those found in the microvasculature [5], [30], [31], [32]. These techniques could be adapted for investigation of the effect of rosette-disrupting agents under shear stress. However, they suffer from various disadvantages, including the requirement to add non-physiological colloidal compounds to increase viscosity that may affect rosette strength [32], and time delays between applying shear stress and rosette assessment by microscopy.

We therefore set out to develop a flow-based rosetting assay in which it would be possible to test the effect of anti-rosetting agents under shear stresses similar to those experienced by IEs in the microvasculature *in vivo* [29], [33]. Using the new assay we tested antibodies against the NTS-DBLα region of PfEMP1 and sulphated glycoconjugates (heparin and fucoidan) against two *P. falciparum* rosetting strains, to determine if anti-rosetting agents show similar rosette disruption activity under physiologically relevant flow conditions compared to those measured in static assays. We successfully established the rosetting flow assay, and were able to quantify the effect of rosette-disrupting agents under shear stresses equivalent to those found *in vivo*.

## Materials and Methods

### Parasite Strains

Two well-characterized laboratory rosetting strains IT/R29 [10], [25] and TM284R+ [24], [34] were used. These two strains have distinct rosetting phenotypes, with TM284R+ showing IgM-positive rosetting (indicating that the rosetting IEs bind the Fc region of non-immune IgM antibodies so that their surface becomes coated with IgM from human plasma) [35], whereas IT/R29 is an IgM-negative rosetting strain [36]. The IgM-positive rosetting phenotype is most closely linked to severe malaria in African children [36]. For both strains, we have previously identified the PfEMP1 variant that mediates rosetting, and raised potent rosette-disrupting antibodies against the NTS-DBLα region of PfEMP1 [10], [24], [25]. In addition, the sensitivity of each strain to sulphated glycoconjugates has also been described, with IT/R29 rosettes being disrupted by both heparin and fucoidan [15], whereas TM284R+ rosettes are not disrupted by heparin and only partially disrupted by fucoidan [16].

### Parasite Culture

Parasite cultures were grown in O+ erythrocytes (Scottish National Blood Transfusion Service) in parasite culture medium, comprising RPMI 1640 medium (Invitrogen) supplemented with 5% pooled human serum (Scottish National Blood Transfusion Service), 2 mM L-glutamine (Invitrogen), 25 mM HEPES (Lonza), 20 mM glucose (Sigma), 25 µg/mL gentamicin gulphate (Lonza) and 0.25% Albumax II (Gibco). The pH was adjusted to 7.2–7.4 with the addition of NaOH (Sigma) and flasks were kept a constant temperature of 37°C and gassed with 1% O_2_/3% CO_2_/96% N_2_. Parasite maturity and parasitaemia were assessed by microscopy of fixed thin blood smears stained with 10% Giemsa. All cultures used were free from mycoplasma contamination [37].

### Staining Parasites for Rosetting Assays

To visualize IEs within rosettes, prior to each assay, the culture was stained with either 25 µg/ml ethidium bromide (Sigma) or 1∶10,000 SyBr Green (Molecular Probes) for 5 minutes at 37°C. The IEs where then washed twice with incomplete media (RPMI 1640 as above, but without human serum or Albumax II), and resuspended at 1% haematocrit in Binding Medium (BM) composed of RPMI-1640 (without bicarbonate) supplemented with 10% human serum, 2 mM L-glutamine (Invitrogen), 25 mM HEPES (Lonza), 20 mM glucose (Sigma) and 25 µg/mL gentamicin sulphate (Lonza). The absence of bicarbonate allows pH to remain stable in non-gassed conditions during the adhesion assays.

### Assessment of Rosette Frequency (RF) in Static Assays

Rosette frequency (RF) was determined by viewing a wet preparation of stained culture suspension (10 µl on a clean microscope slide covered by a 22×22 mm coverslip) using simultaneous white light and fluorescence to visualize both infected and uninfected erythrocytes (Leica DMLB2 microscope). A rosette is defined as an IE that binds two or more uninfected erythrocytes. The RF is the percentage of IEs forming rosettes out of 200 IEs counted. In each independent static experiment, RF was calculated in three separate wet preparations from each sample, and a mean value determined from the triplicate counts.

### Rosette Disruption Assays under Static Conditions

Rosette disruption assays were carried out with parasite cultures of greater than 50% rosette frequency. Parasite cultures were pre-stained with 25 µg/ml of ethidium bromide as described above. Rabbit polyclonal antibodies (total purified IgG) against the NTS-DBLα region of the ITvar09/R29var1 [25] and TM284var1 [24] PfEMP1 variants were added to the corresponding IE suspension to give a final concentration of 100, 10 and 1 µg/ml, and incubated at 37°C for 30 minutes and compared to a “no additive” negative control. Similarly, heparin and fucoidan (Sigma) were added to give final concentrations of 100, 10 and 1 µg/ml (heparin) and 100 µg/ml (fucoidan), and incubated at 37°C for 30 minutes and compared to a “no additive” negative control. Rosette frequency was assessed as above.

### Rosette Disruption Assays under Flow

Flow assays were carried out using the ibidi flow system (ibidi, Germany, http://ibidi.com/xtproducts/en/Instruments-Accessories/Pump-Systems/ibidi-Pump-System). This consists of a pump and fluidic unit used with a perfusion set (ibidi yellow/green: Cat. No. #10964– length 50 cm, inner diameter 1.6 mm with 10 ml reservoirs) and a µ-slide I 0.8 (dimensions: 800 µm×5 mm×50 mm) constructed of optical quality polymer. The flow rates tested gave a wall shear stress of 0.5, 0.75 and 1 dyn/cm^2^, which have been widely used to mimic wall shear stresses in the microvasculature [33], [38], [39], [40]. All observations and measurements of rosetting under physiological shear stress where conducted within the area of homogenous shear stress. For µ-slide I 0.8, this is at least 800 µm away from the channel walls, however, as recommended by the manufacturer, all observations are made down the central line of the slide, away from the 800 µm boundary from the channel wall (Application Note AN-11, www.ibidi.de). The μ-slide I chambers (ibidi, Germany) were blocked overnight at 4°C with PBS/1% BSA. In the absence of this blocking step, both IEs and non-IEs showed some non-specific adhesion to the chamber wall. The following day, 3 ml of pre-stained IE suspension at 5% parasitaemia and 1% Ht in BM (pH 7.4)/10% pooled human serum was prepared. The suspension was added to the perfusion system and allowed to flow over the μ-slide I for 5 minutes at 0.5 dyn/cm^2^, before stopping the flow and immediately capturing both bright-field and fluorescence images from 10 consecutive fields (0.2 mm^2^ per field) with a 20× objective, using an EVOS FL digital inverted fluorescence microscope (AMG). The flow was restarted at 5 dyn/cm^2^ for 2 minutes to disperse any cells that may have settled in the reservoirs, before setting the flow rate to 0.75 dyn/cm^2^ and repeating the procedure as above. After capturing images at 0.75 dyn/cm^2^, a further 2 minute dispersal step at 5 dyn/cm^2^was carried out, and the above procedure repeated at 1 dyn/cm^2^. For assessment of rosetting in the presence of rosette-disrupting agents, the relevant amount of drug/antibody was added to the parasite suspension 30 minutes prior to the assay, then the suspension was flowed over the chamber slide at the shear stresses described above. In each experiment, the “no antibody/drug” negative control sample was tested first, followed by the samples containing antibody/drug starting from the lowest concentration (1 µg/ml) followed by 10 µg/ml then 100 µg/ml.

### Quantification of Rosetting Under Flow

Using ImageJ software (http://rsb.info.nih.gov/ij/), each of the 10 images corresponding to 10 fields per sample were analysed, and the total rosette area per field was measured. The composite images (containing both bright-field and fluorescence information) were opened in ImageJ, and as the EVOS FL software automatically adds a scale bar to the composite image, this served as a marker to calibrate the images for measurement. Using the existing scale bar, it was determined that 2.56 pixels = 1 µm. With the scale set, the images could then be prepared for analysis. In preparation for measurement, the threshold settings were adjusted to highlight the rosettes, in order to allow the margins of the cell clusters to be identified more clearly. Briefly, the captured images were processed using brightness and contrast, followed by threshold to enhance the visualization of rosettes against the background. Then, using the Analyze/Measurement settings, rosettes were selected with the ‘wand’ tool, which highlights the circumference of the selected rosette and calculates the area (µm^2^). All of the measurements from each image were added and the results expressed as total rosette area (µm^2^) from 10 fields.

### Statistical Analysis and Graphing

Data were analysed and graphs prepared using GraphPad Prism v5.0. (GraphPad Software Inc.) and JMP v10.0.2 (SAS Institute Inc.). Mean rosetting values from at least three independent experiments for each antibody/drug and shear stress were compared by one-way ANOVA, with p<0.05 being taken as statistically significant. The significant tests were analysed further by Tukey’s post-hoc test, to compare individual antibody/drug concentrations to the control. Finally, rosetting values were converted to percentage of the “no additive” control value, to allow direct comparison between data from static and flow assays. In a small number of cases, the assumption of homogeneity of variance was violated, however, repeating the ANOVA analysis with Welch’s F test and Dunnett’s post-hoc test did not materially alter the results.

## Results

### Large “Multi-rosettes” form under Flow Conditions

**Figure 1:**
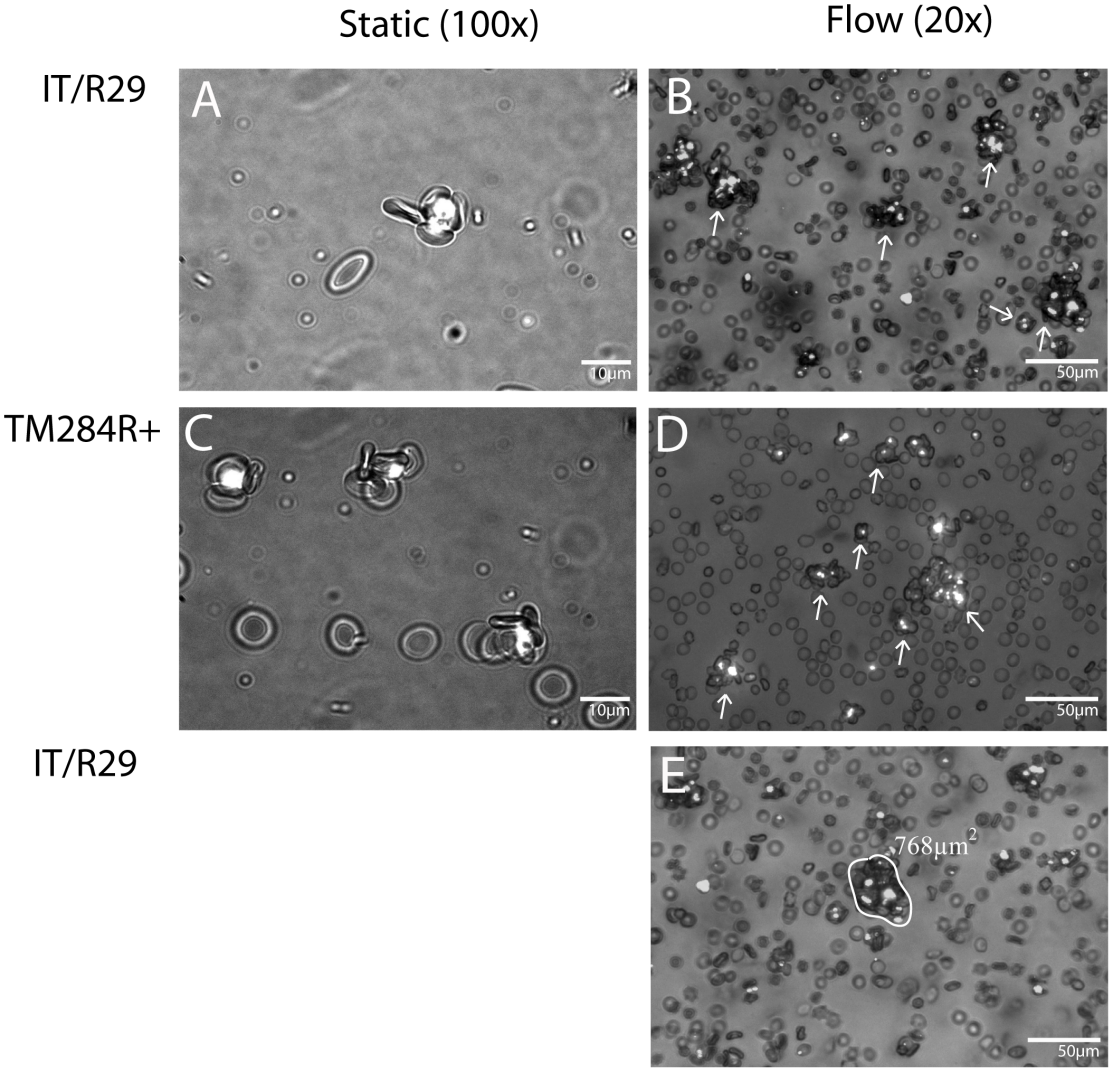
Rosetting in static and flow conditions. A**)** Parasite strain IT/R29 under static conditions. IEs at 5% parasitaemia and 1% haematocrit were stained with 25 µgml ethidium bromide to visualize the parasite DNA, seen as bright white areas within IEs. Wet preparations were viewed under dual brightfield/UV fluorescence (×100). The images were captured using a Q-imaging camera attached to an Olympus BX50 microscope. B) Parasite strain IT/R29 under flow. 3 ml of parasite culture at 5% parasitaemia and 1% haematocrit was flowed over a µ-slide I (ibidi, Germany) at 0.5 dyn/cm^2^ for 5 minutes. The flow was stopped and images captured immediately using an EVOS FL inverted fluorescence microscope (×20). IEs were stained with 1∶10,000 dilution of SyBr Green prior to the assay to identify IEs within the rosettes clearly. White arrows indicate “multi-rosettes” seen under flow conditions. C**)** Parasite strain TM284R+ under static conditions, as described in A. D) Parasite strain TM284R+ under flow as described in B. E) IT/R29 multi-rosette from a flow experiment at 0.5 dyn/cm^2^, demonstrating the use of ImageJ to draw the circumference and measure the surface area of the selected multi-rosette and expressed as µm^2^.

To determine the ability of IEs to form rosettes under shear stresses similar to those experienced in the microvasculature *in vivo*, two *P. falciparum* rosetting strains IT/R29 and TM284R+ were examined using an ibidi perfusion system at 0.5, 0.75 and 1 dyn/cm^2^. In each case, the rosette frequency (percentage of IEs binding two or more uninfected erythrocytes) of the culture under static conditions was assessed prior to the flow experiments. Under static conditions, rosettes are usually small, isolated clusters of cells, with one or two IE and 2–10 non-IE per rosette (Fig. 1A and C). In contrast, under flow, many “multi-rosettes” are formed due to individual rosettes sticking together into larger clumps (Fig. 1B and D, white arrows). These large multi-rosettes behaved as single ‘objects’ within the flow (see Video S1). The experiments described below focus on two well-characterized rosetting parasite strains IT/R29 and TM284R+. However, several other rosetting *P. falciparum* strains (IT/PAR+, TM180R+, Muz12R+ and HB3R+) were also examined under flow, and in all cases, large “multi-rosettes” formed under physiological flow conditions. Non-IEs and non-rosetting *P. falciparum* cultures did not form rosettes or multi-rosettes under flow.

Because of the presence of multi-rosettes comprising three-dimensional clumps with many IEs buried within the clump, it was not possible to count rosette frequency (percentage of IEs in rosettes) in the flow assay, as is routinely done in the static assay. Therefore, an alternative method of quantification of rosetting was developed, using image analysis software (ImageJ). This software allows for measurement of the area (µm^2^) of every cell cluster in a field (0.2 mm^2^), including both individual rosettes and multi-rosettes, as described in detail in the Methods section. Briefly, the wand tool was used to highlight the circumference of the selected rosette and calculate the area (µm^2^) (Fig. 1E). All of the rosette area measurements from each image were then added to give the total rosette area (µm^2^) from 10 fields at every shear stress and condition examined below. In all experiments the multi-rosettes contained IEs, and similar clusters of non-IEs were not seen.

### Rosetting does not Vary Over the Range of Shear Stresses from 0.5–1 dyn/cm^2^

**Figure 2:**
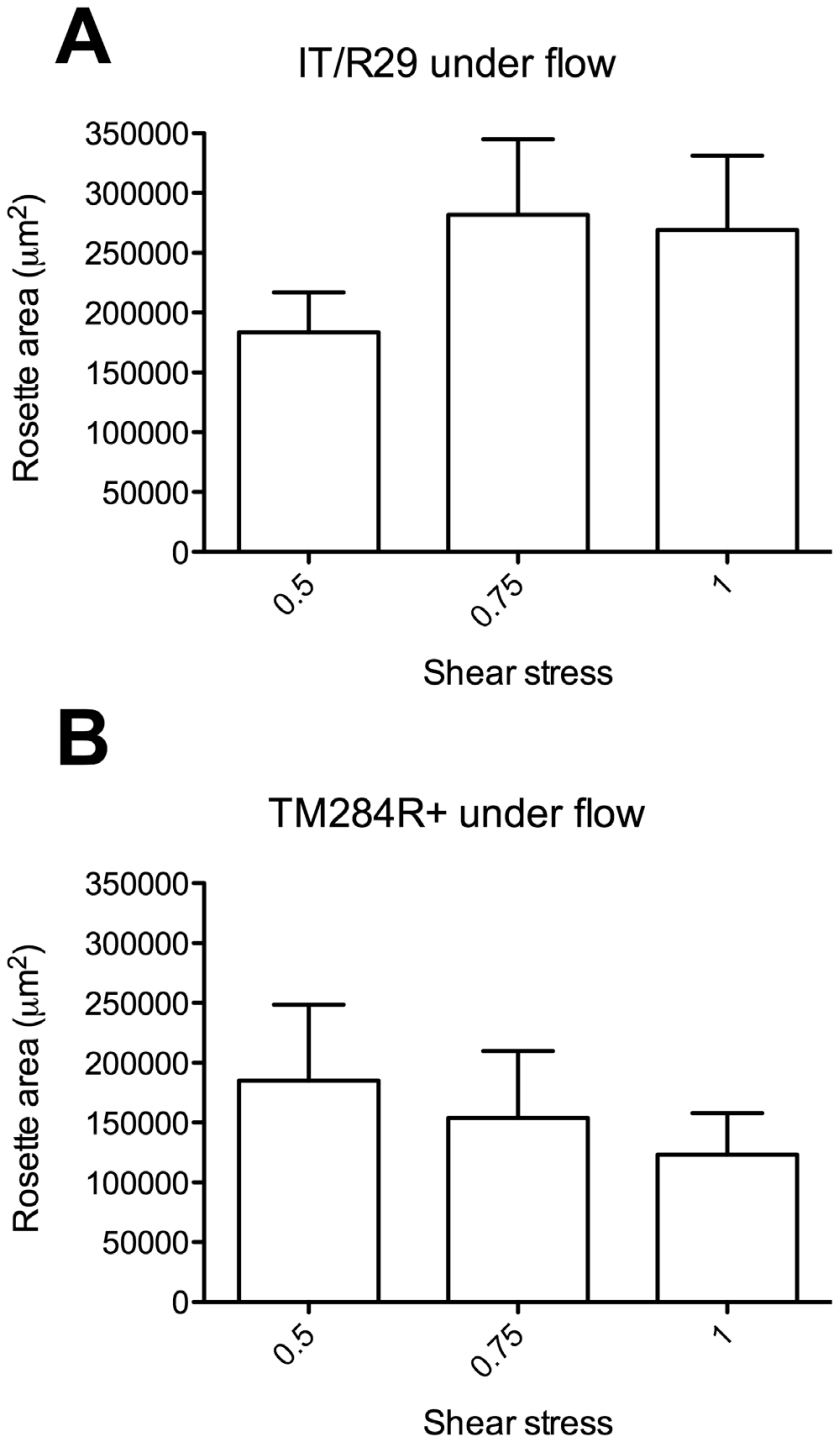
The effect of shear stress (0.5–1 dyn/cm^2^) on rosetting. Parasite cultures were subjected to shear stresses of 0.5, 0.75 or 1.0/cm^2^ in an ibidi flow system as described for Figure 1, and images from 10 fields (20× objective) were captured by fluorescence microscopy. The total rosette area (µm^2^) from 10 fields at each shear stress was determined using ImageJ software. A) Parasite strain IT/R29 was tested in six independent experiments, and the mean of the total rosette area from each experiment is shown. Error bars indicate standard error of the mean. B) Parasite strain TM284R+ was tested in four independent experiments, and the mean of the total rosette area from each experiment is shown. Error bars indicate standard error of the mean. There were no statistically significant differences between the mean rosette areas at the three different shear stresses for either parasite strain by one-way ANOVA.

We firstly investigated whether rosetting was affected by shear stress between the range of 0.5 and 1 dyn/cm^2^. These values were chosen because they have been widely used to approximate the shear stress in post-capillary venules in previous studies of *P. falciparum* IE cytoadherence to endothelial cells [33], [38], [39], [40]. Using *P. falciparum* strain IT/R29, there was a trend towards higher rosetting at higher shear stress, however, this was not statistically significant (Fig. 2A, one-way ANOVA F(2,15) = 1.0, p = 0.40, n = 6 experiments). Likewise, with parasite strain TM284R+ there was no statistically significant effect of shear stress in the range 0.5–1 dyn/cm^2^ on rosetting, although in this case there was a trend towards reduced rosetting with increasing shear stress (Fig. 2B, one-way ANOVA F(2,9) = 0.3, p = 0.72, n = 4 experiments).

### Effects of Anti-rosetting Agents on IT/R29 Parasites under Flow

**Figure 3:**
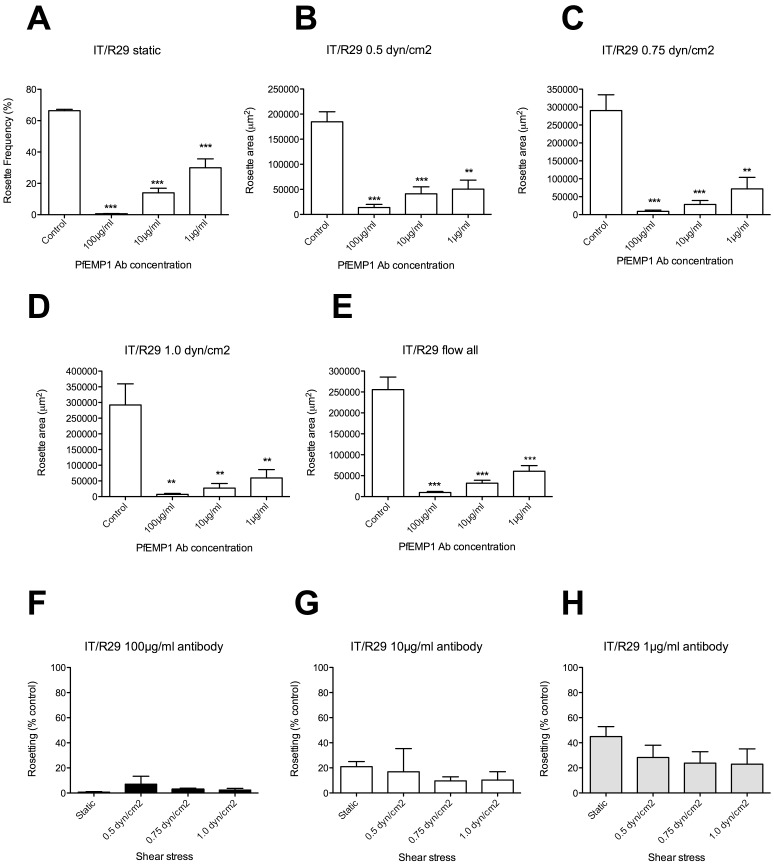
The effect of PfEMP1 antibodies on rosetting in static and flow assays with parasite strain IT/R29. Antibodies to the NTSDBLα region of the PfEMP1 variant ITvar09/R29var1 were added to IT/R29 parasite culture suspension at a final concentration of 100, 10 and 1 µg/ml of antibody and incubated for 30 mins before assessment of rosetting. The control was a culture suspension with no added antibody. A) Static assay: the percentage in IEs in rosettes (rosette frequency) was assessed by fluorescence microscopy of ethidium bromide-stained wet preparations. B–D) Flow assays: total rosette area (µm^2^) from 10 fields (20× objective) was determined after 5 minutes under flow at each shear stress using ImageJ software. E) Flow assays: experiments at all shear stresses combined. F–H) To allow direct comparison of the results from static and flow assays, the rosetting values from each experiment were converted to a percentage of the “no additive” control value. F) IT/R29 parasites with 100 µg/ml PfEMP1 antibody. G) IT/R29 parasites with 10 µg/ml PfEMP1 antibody. H) IT/R29 parasites with 1 µg/ml PfEMP1 antibody. Mean and standard error from three independent experiments (A–D and F–H) or nine experiments (E) are shown. Mean values that are statistically significantly different from the control value (A–E) or the static value (F–H) by one-way ANOVA with Tukey’s post test are shown by asterisks, **p<0.01, ***p<0.001.

Having confirmed that rosettes do persist under physiologically-relevant shear stresses (Fig. 1), and can be quantified under flow conditions (Fig. 2), we proceeded to test the effect of anti-rosetting agents under flow. We previously showed that antibodies against the NTS-DBLα region of the rosette-mediating PfEMP1 variant ITvar09 (also known as R29var1) are able to significantly disrupt rosettes in the IT/R29 parasite strain under static conditions [25]. To determine if shear stress affects the rosette-disrupting ability of these antibodies, we pre-incubated IT/R29 culture suspension with varying concentrations of antibody (1, 10 and 100 µg/ml) for 30 minutes prior to measuring rosetting in a static assay and under flow at 0.5, 0.75 and 1 dyn/cm^2^. In each case rosetting was compared to a control culture with no added antibody. As expected, all three concentrations of antibody gave significant rosette disruption in the static assay (Fig. 3A, one-way ANOVA F(3,8) = 78.3, p<0.0001). The same result was seen under flow, with significant rosette disruption at all three antibody concentrations at all three shear stresses (Fig. 3B, 0.5 dyn/cm^2^, one-way ANOVA F(3,8) = 24.4, p = 0.0002; Fig. 3C, 0.75 dyn/cm^2^, one-way ANOVA F(3,8) = 21.6, p = 0.0003 and Fig. 3D, 1.0 dyn/cm^2^, one-way ANOVA F(3,8) = 12.7, p = 0.002). Because the data in Figure 2 showed that there were no statistically significant differences between the different shear stresses, we also examined the data after combining the experiments performed at 0.5, 0.75 and 1.0 dyn/cm^2^. As expected, significant rosette disruption at all three antibody concentrations was seen (Fig. 3E, one-way ANOVA F(3,32) = 45.1, p<0.0001). To determine if the extent of rosette disruption in the flow assays differed from that in the static assay, all data were converted into percentage of the “no antibody” control, and the flow values at each antibody concentration were compared to the static value (Fig. 3F–H). At all three antibody concentrations, there were no statistically significant differences between the rosetting values derived from the flow assays compared to the static assay.

**Figure 4:**
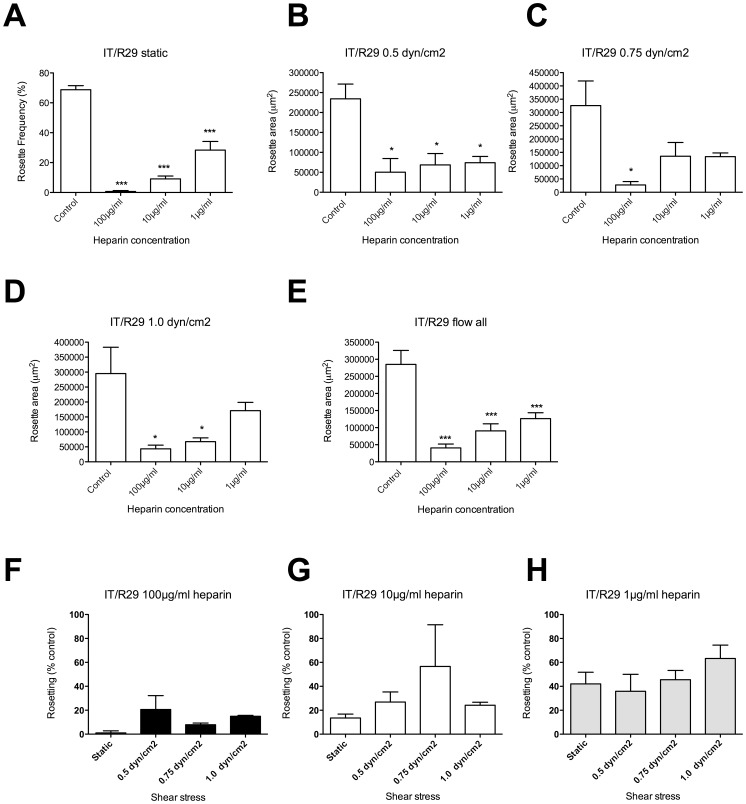
The effect of heparin on rosetting in static and flow assays with parasite strain IT/R29. Heparin was added to IT/R29 parasite culture suspension at a final concentration of 100, 10 and 1 µg/ml and incubated for 30 mins before assessment of rosetting. The control was a culture suspension with no added heparin. A) Static assay: the percentage in IEs in rosettes (rosette frequency) was assessed by fluorescence microscopy of ethidium bromide-stained wet preparations. B–D) Flow assays: total rosette area (µm^2^) from 10 fields (20× objective) was determined after 5 minutes under flow at each shear stress using ImageJ software. E) Flow assays: experiments at all shear stresses combined. F–H) To allow direct comparison of the results from static and flow assays, the rosetting values from each experiment were converted to a percentage of the “no additive” control value. F) IT/R29 parasites with 100 µg/ml heparin. G) IT/R29 parasites with 10 µg/ml heparin. H) IT/R29 parasites with 1 µg/ml heparin. Mean and standard error from three independent experiments (A–D and F–H) or nine experiments (E) are shown. Mean values that are statistically significantly different from the control value (A–E) or the static value (F–H) by one- way ANOVA with Tukey’s post test are shown by asterisks, *p<0.05, ***p<0.001.

The same procedure was followed with a second, distinct rosette-disrupting agent, by testing the effect of heparin on IT/R29 rosetting at three concentrations (1, 10 and 100 µg/ml) under static and flow conditions. In each case rosetting was compared to a control culture with no added heparin. Under static conditions, IT/R29 rosetting was significantly reduced at all three heparin concentrations (Fig. 4A, one-way ANOVA F(3,8) = 81.1, p<0.0001). Similar results were seen under flow, with significant disruption of rosetting at all three shear stresses (Fig. 4B, 0.5 dyn/cm^2^ one-way ANOVA F(3,8) = 8.1, p = 0.0082; Fig. 4C, 0.75 dyn/cm^2^ one-way ANOVA F(3,8) = 5.3, p = 0.03 and Fig. 4D, 1.0 dyn/cm^2^ one-way ANOVA F(3,8) = 6.0, p = 0.02). However, the high level of variation between experiments under flow meant that not all individual heparin concentrations were statistically significant (Fig. 4C, 10 and 1 µg/ml and Fig. 4D, 1 µg/ml are not statistically different to the control value by Tukey’s post test). When the data from experiments at all three shear stresses were combined, all concentrations of heparin gave significantly reduced rosetting (Fig. 4E, one-way ANOVA F(3,32) = 17.8, p = <0.0001). As above, conversion of all data to a percentage of the “no additive” control value was carried out to allow direct comparison between results from the static and flow assays. At all three heparin concentrations, there were no statistically significant differences between the rosetting values derived from the flow assays compared to the static assay (Fig. 4F–H).

IT/R29 was also tested in static and flow assays with 100 µg/ml fucoidan, which completely disrupted all rosettes in every experiment. Representative images from this, and the above rosetting flow experiments are shown in Fig. S1.

### Effects of Anti-rosetting Agents on TM284R+ Parasites under Flow

**Figure 5:**
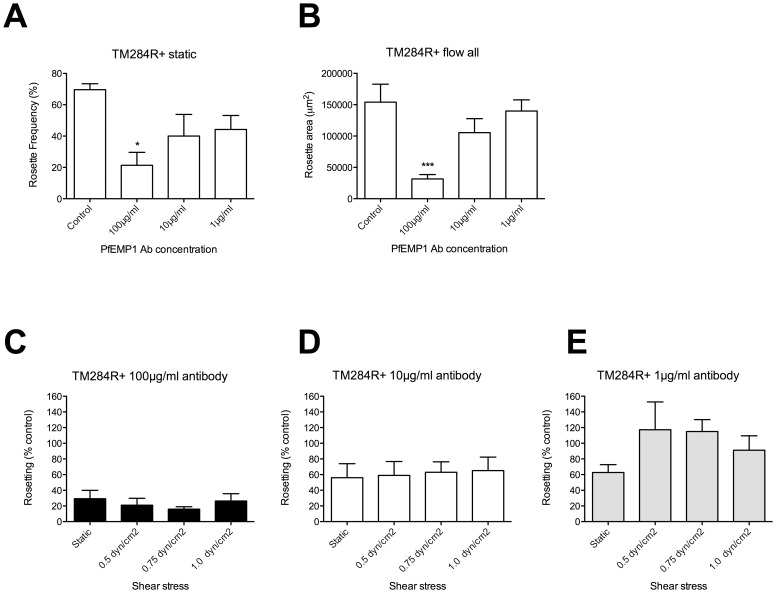
The effect of PfEMP1 antibodies on rosetting in static and flow assays with parasite strain TM284R+. Antibodies to the NTSDBLα region of the PfEMP1 variant TM284var1 were added to TM284R+ parasite culture suspension at a final concentration of 100, 10 and 1 µg/ml of antibodies and incubated for 30 mins before assessment of rosetting. The control was a culture suspension with no added antibody. A) Static assay: the percentage in IEs in rosettes (rosette frequency) was assessed by fluorescence microscopy of ethidium bromide-stained wet preparations. B) Flow assay using combined data from experiments at 0.5, 0.75 and 1.0 dyn/cm^2^. The total rosette area (µm^2^) from 10 fields (20× objective) was determined after 5 minutes under flow at each shear stress using ImageJ software. C–E) To allow direct comparison of the results from static and flow assays, the rosetting values from each experiment were converted to a percentage of the “no additive” control value. C) TM284R+ parasites with 100 µg/ml PfEMP1 antibody. D) TM284R+ parasites with 10 µg/ml PfEMP1 antibody. E) TM284R+ parasites with 1 µg/ml PfEMP1 antibody. Mean and standard error from four independent experiments (A and C–E), or twelve experiments (B) are shown. Mean values that are statistically significantly different from the control value (A–B) or the static value (C–E) by one-way ANOVA with Tukey’s post test are shown by asterisks, *p<0.05.

Further experiments were carried out with a different *P. falciparum* rosetting strain TM284R+, whose rosettes are more resistant to rosette-disrupting agents than IT/R29 [16]. Antibodies to the NTS-DBLα region of the rosette-mediating PfEMP1 variant TM284var1 were used [24], and tested in static assays and under three different shear stresses as above. In the static assay, statistically significant rosette disruption was seen at 100 µg/ml of antibody only (Fig. 5A, one-way ANOVA F(3,12) = 4.5, p = 0.02). Similar trends of rosette disruption at 100 µg/ml of antibody were seen in the flow assay at all three shear stresses (Fig. S2 A–C). However, due to the high level of variation in the control values between experiments, these differences were not statistically significant (Fig. S2A, 0.5 dyn/cm^2^, one-way ANOVA F(3,12) = 2.1, p = 0.15; Fig. S2B, 0.75 dyn/cm^2^, one-way ANOVA F(3,12) = 2.3, p = 0.12; Fig. S2C, 1.0 dyn/cm^2^, one-way ANOVA F(3,12) = 2.1, p = 0.16). When the data from experiments at all three shear stresses were combined, 100 µg/ml of antibody gave significantly reduced rosetting compared to the control (Fig. 5B, one-way ANOVA F(3,32) = 7.1, p = 0.0005). When data were converted to a percentage of the “no antibody” control as above, there were no statistically significant differences between rosetting values derived from the flow assays compared to the static assay (Fig. 5 C–E).

**Figure 6:**
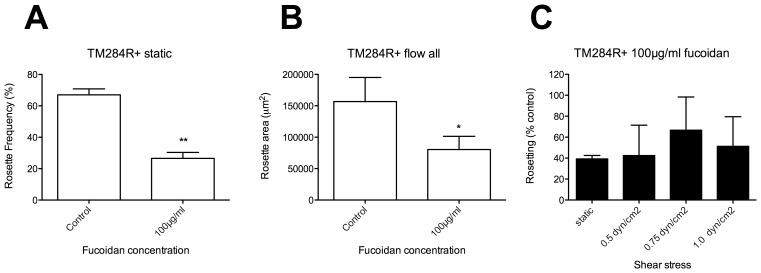
The effect of fucoidan on rosetting in static and flow assays with parasite strain TM284R+. Fucoidan was added to TM284R+ parasite culture suspension at a final concentration of 100 µg/ml and incubated for 30 mins before assessment of rosetting. The control was a culture suspension with no added fucoidan. A) Static assay: the percentage in IEs in rosettes was assessed by fluorescence microscopy of ethidium bromide-stained wet preparations. B) Flow assay using combined data from experiments at 0.5, 0.75 and 1.0 dyn/cm^2^. The total rosette area (µm^2^) from 10 fields (20× objective) was determined after 5 minutes under flow at each shear stress using ImageJ software. C) To allow direct comparison of the results from static and flow assays, the rosetting values from each experiment were converted to a percentage of the “no additive” control value. Mean and standard error from three independent experiments (A and C) or nine experiments (B) are shown. Mean values that are statistically significantly different from the control value (A–B) or the static value (C) by a two-tailed paired t test are shown by asterisks, *p<0.05, **p<0.01.

TM284R+ rosettes have been shown previously to be resistant to heparin in static assays, but are partially disrupted by fucoidan at 100 µg/ml [16]. We therefore tested the ability of 100 µg/ml fucoidan to disrupt TM284R+ rosettes in static and flow conditions. As expected, in the static assay the TM284R+ rosettes were significantly disrupted by 100 µg/ml fucoidan (Fig. 6A, two-tailed paired t test, p = 0.0049). A similar pattern of disruption was seen under flow (Fig. S3 A–C), although this only reached statistical significance when experiments from all three shear stresses were combined (Fig. 6B, two-tailed paired t test, p = 0.04). When data at each shear stress were converted to a percentage of the “no antibody” control as above, there were no statistically significant differences between rosetting values derived from the flow assays compared to the static assay (Fig. 6C).

The effect of 100 µg/ml heparin on TM284R+ was also tested in a single experiment, but as expected from previous work, high levels of rosetting were seen in all samples with no evidence of any rosette disruption. This was not formally quantified, however, representative images from this, and the above rosetting flow experiments are shown in Fig. S4.

## Discussion

This study developed a new *P. falciparum* rosetting flow assay and investigated the effect of anti-rosetting agents under physiological shear stresses compared to static assays. Current screening methods for anti-rosetting agents use static wet preparations and the IEs are not subjected to shear stresses similar to those that would be experienced *in vivo*. We hypothesized that the effect of rosette-disrupting agents might be enhanced when exposed to physiologically relevant shear stresses similar to those found within human capillaries and post-capillary venules (0.5–1.0 dyn/cm^2^), because the shear stresses might contribute to the disruption of the cell clusters.

In order to test our hypothesis, we established a new rosetting flow assay using an ibidi flow system and ImageJ software to quantify rosetting under different shear stresses. The ibidi perfusion system allows for the control of shear stress in a closed system, while using a relatively small volume (3 ml) of culture suspension. The ibidi µ-slide I chambers allow for the visualisation of rosettes under flow by digital inverted microscopy and the capturing of images for later analysis. The system allows for capturing of images of rosettes directly from flow, without the need for intermediate steps.

Using this system, we found that, in agreement with previous work [5], [30], [31], [32], rosettes do exist under physiologically relevant shear stresses (Figs. 1 and 2). Once subjected to flow, classical rosettes comprising one IE plus two or more non-IEs (Fig. 1 A and C) often combine with other rosettes and non-IEs to form larger clusters of “multi-rosettes” (Fig. 1 B and D). These behave within the flow as a single object and have various sizes and conformations (Video S1). Large multi-rosettes (sometimes called “giant rosettes”) have occasionally been reported from static assays [41]. Our experiments show that multi-rosettes occur commonly under the continuous flow conditions used here.

Using the new flow assay, we tested rosette-disrupting PfEMP1antibodies and sulphated glycoconjugate drugs on two *P. falciparum* rosetting strains (IT/R29 and TM284R+), and compared the results from static and flow assays. In all cases we found very similar patterns of rosette disruption under flow compared to static conditions (Figs. 3–6). There was no evidence to support any statistically significant difference between the results of the flow and static assays at the antibody and drug concentrations tested. Therefore under the conditions used here, the data do not support our hypothesis, but instead suggest that physiologically relevant shear stresses neither enhance nor reduce the activity of rosette-disrupting agents such as PfEMP1 antibodies or sulphated glycoconjugate compounds. Whether other rosette-disrupting interventions, such as soluble recombinant rosetting receptors including CR1 [10], [42] and ABO blood group sugars [9], [43] will behave similarly under flow will require further investigation.

The new rosetting flow assay described here allows for the assessment of rosetting under shear stresses similar to those found *in vivo*. However, the assay does have some limitations and disadvantages. The flow assay is time-consuming, requires specialized equipment, and shows more intrinsic variation from one experiment to the next than the static assay. The most time-consuming part of the procedure is the image analysis, which took several hours per sample, compared to just a few minutes per sample to assess rosetting in the static assay. However, the image analysis could potentially be automated [44], thus stream-lining the process and facilitating more repeated experiments to overcome the innate variation between experiments. The experimental set-up could also be adjusted for future work. In the set of experiments reported here, the rosette-disrupting agents were added prior to addition of the culture into the perfusion system. It would also be possible to add the agents to the parasite culture suspension once it is circulating in the perfusion system to try to make the assay more “life-like”. However, *in vivo*, rosettes do not circulate freely in the peripheral blood, but accumulations of IE and non-IE are seen in post-capillary venules as part of the sequestration process [5], [45], [46]. Rosetting IE may bind directly to the endothelium [47] or may bind to sequestered non-rosetting IE that are adherent to blood vessel walls. A more realistic rosetting assay might involve allowing rosettes to form on endothelial cells or on monolayers of IE bound to a microscope slide. In addition, further modifications could be carried out in an attempt to mimic in vivo conditions more closely, such as carrying out experiments using whole blood, which would include leucocytes, platelets and higher plasma concentrations compared to the existing assay.

Despite the time-consuming nature of the image analysis in the new flow assay, it does have some advantages compared to methods previously used to assess rosetting under shear stresses, including the plate-cone viscometer [30], [31] and LORCA [32]. These assays, whilst subjecting rosettes to shear stress, ultimately calculate rosette frequency from a standard wet preparation. In other words, after applying the shear stress, the culture suspension is removed from the device and then a wet preparation is made and viewed by microscopy. This necessitates a time delay in which rosettes could reform or fall apart, and also necessitates the passing of the cells through a pipette tip, which can apply an inconsistent and poorly defined shear stress to the suspension. The use of a closed perfusion system allows for visualization and data capture of rosettes directly from flow.

## Conclusions

In summary, the experiments shown here confirm that *P. falciparum* rosettes exist under physiologically relevant shear stresses, and describe a new rosetting flow assay that may be useful for researchers interested in malaria host-parasite interactions. There were no statistically significant differences in the rosette-disrupting effects of PfEMP1 antibodies and sulphated glycoconjugates under static and flow conditions. These results increase confidence that the widely-used, quick and simple static rosetting assay is a good predictor of results obtained under physiological shear forces. Therefore static assays are likely to continue to be used for the majority of rosetting experiments and screening assays, while the flow assay may be useful for more detailed analysis of potential rosette-disrupting interventions.

## Supporting Information

Figure S1 **Effect of anti-rosetting agents on IT/R29 rosetting under flow.** Representative images are shown for each rosetting flow experiment.
(TIFF)

Figure S2 **The effect of PfEMP1 antibodies on rosetting in flow assays with parasite strain TM284R+.** Antibodies to the NTS-DBLα region of the PfEMP1 variant TM284var1 were added to TM284R+ parasite culture suspension at a final concentration of 100, 10 and 1 µg/ml of antibody and incubated for 30 mins before assessment of rosetting at A) 0.5 dyn/cm^2^ B) 0.75 dyn/cm^2^ and C) 1.0 dyn/cm^2^. The control was a culture suspension with no added antibody. The total rosette area (µm^2^) from 10 fields (20× objective) was determined after 5 minutes under flow at each shear stress using ImageJ software. Mean and standard error from four independent experiments at each shear stress are shown. No statistically significant differences were found by one-way ANOVA. The static assay data and the pooled flow data from this experiment are shown in Figure 5 of the main manuscript.
(TIFF)

Figure S3 **The effect of fucoidan on rosetting in flow assays with parasite strain TM284R+.** 100 µg/ml of fucoidan was added to TM284R+ parasite culture suspension and incubated for 30 mins before assessment of rosetting at A) 0.5 dyn/cm^2^ B) 0.75 dyn/cm^2^ and C) 1.0 dyn/cm^2^. The control was a culture suspension with no added fucoidan. The total rosette area (µm^2^) from 10 fields (20× objective) was determined after 5 minutes under flow at each shear stress using ImageJ software. Mean and standard error from three independent experiments at each shear stress are shown. No statistically significant differences were found by one-way ANOVA. The static assay data and the pooled flow data from this experiment are shown in Figure 6 of the main manuscript.
(TIFF)

Figure S4 **Effect of anti-rosetting agents on TM284R+ rosetting under flow.** Representative images are shown for each rosetting flow experiment.
(TIF)

Video S1 **The** ***P. falciparum*** **rosetting strain IT/PAR+ was added to the perfusion system at 5% parasitemia and 1% haematocrit and subjected to a shear stress 0.5** **dyn/cm^2^.** Video was captured of the cells in flow (×20 objective) using a Leica 6000B inverted microscope equipped with DFC360FX camera recording at 30 frames per second. The video clip shows IT/PAR+ “multi-rosettes” under flow and how they exist as single objects, and further demonstrates the various sizes and conformations observed under flow conditions.
(MOV)
