# Supplementary figures and images for: The Effect of Anti-Rosetting Agents against Malaria Parasites under Physiological Flow Conditions
Source: PLoS One. 2013 Sep 16;8(9):e73999. doi: 10.1371/journal.pone.0073999 (PMC3774797; doi:10.1371/journal.pone.0073999)

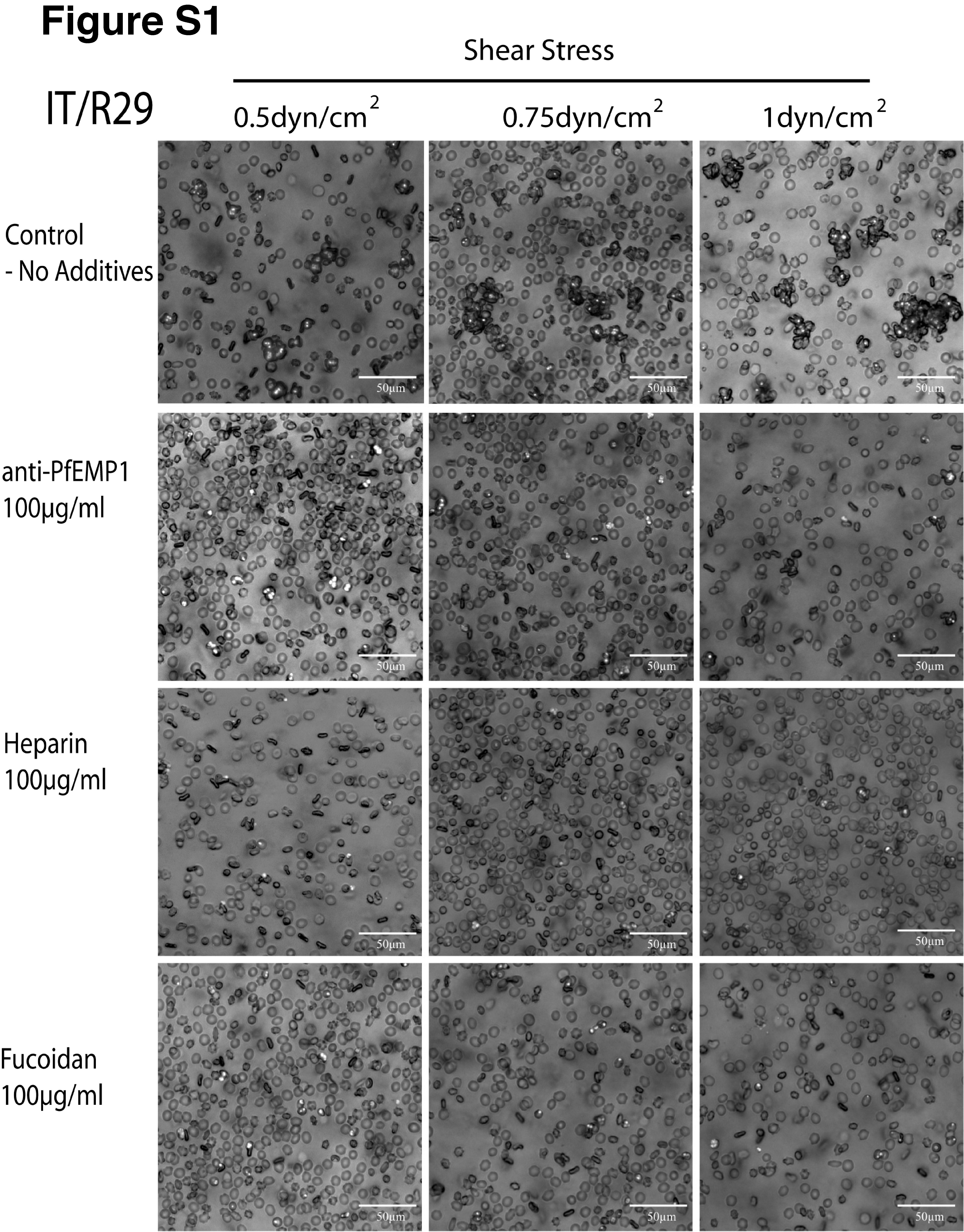

Supplement: Figure S1 — Effect of anti-rosetting agents on IT/R29 rosetting under flow. Representative images are shown for each rosetting flow experiment. (TIFF) [file pone.0073999.s001.tiff]

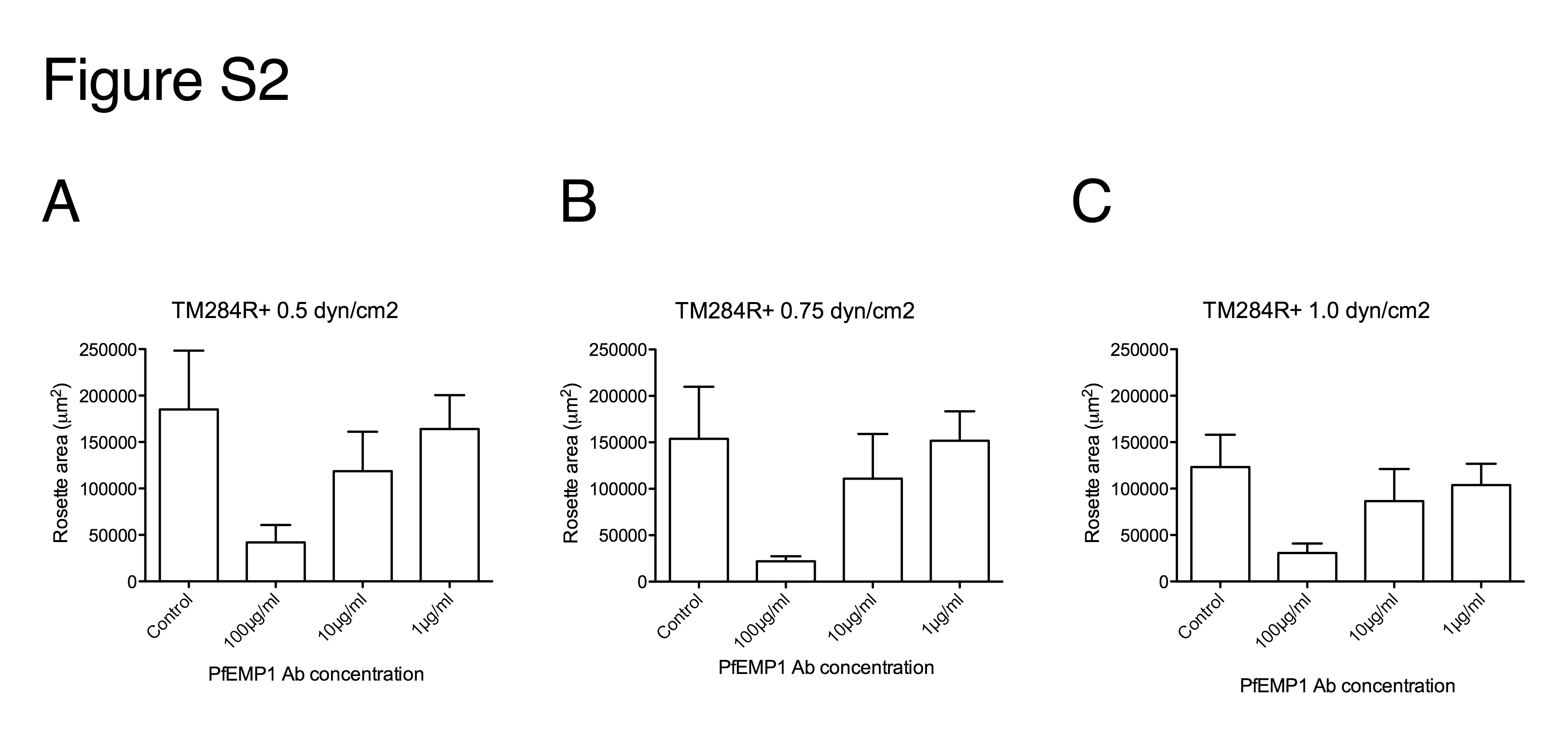

Supplement: Figure S2 — The effect of PfEMP1 antibodies on rosetting in flow assays with parasite strain TM284R+. Antibodies to the NTS-DBLα region of the PfEMP1 variant TM284var1 were added to TM284R+ parasite culture suspension at a final concentration of 100, 10 and 1 µg/ml of antibody and incubated for 30 mins before assessment of rosetting at A) 0.5 dyn/cm2 B) 0.75 dyn/cm2 and C) 1.0 dyn/cm2. The control was a culture suspension with no added antibody. The total rosette area (µm2) from 10 fields (20× objective) was determined after 5 minutes under flow at each shear stress using ImageJ software. Mean and standard error from four independent experiments at each shear stress are shown. No statistically significant differences were found by one-way ANOVA. The static assay data and the pooled flow data from this experiment are shown in Figure 5 of the main manuscript. (TIFF) [file pone.0073999.s002.tiff]

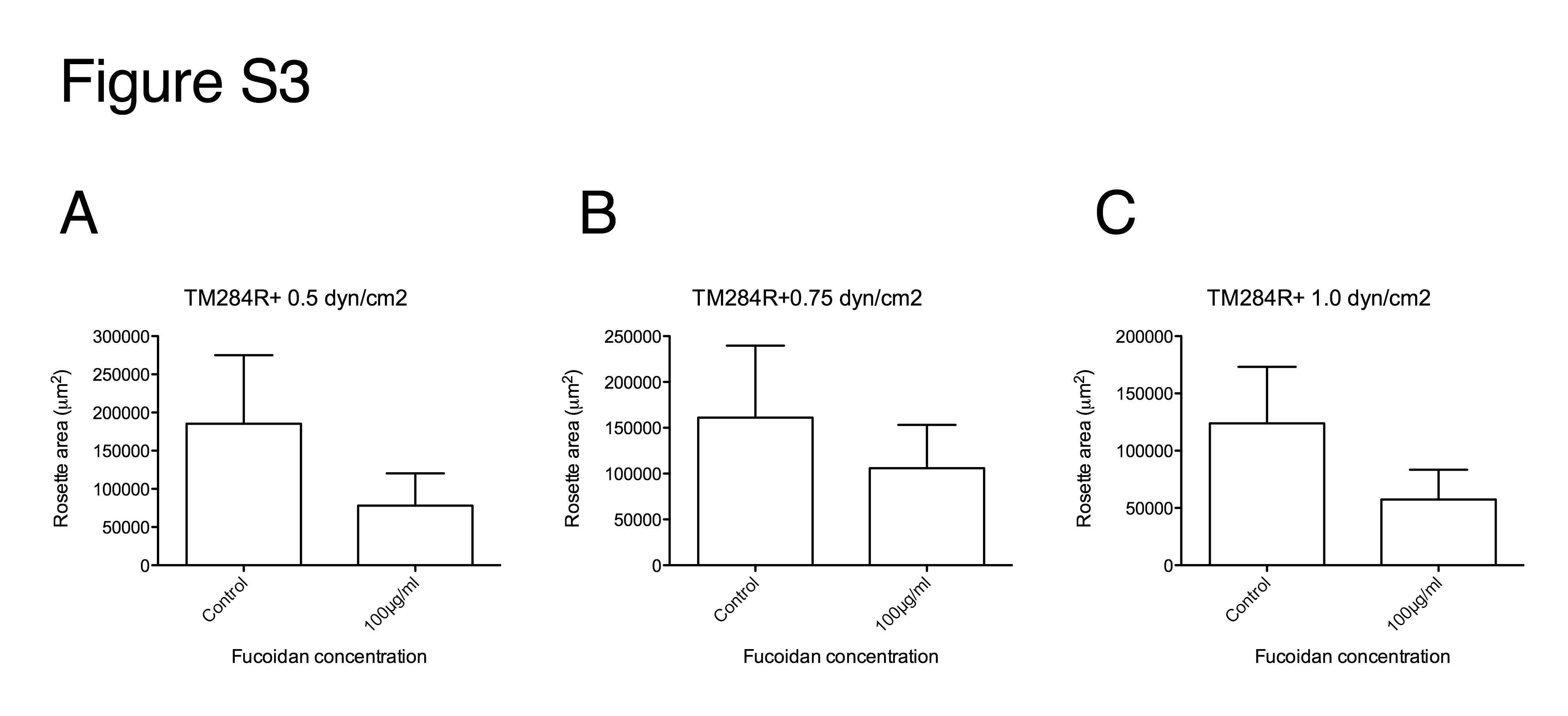

Supplement: Figure S3 — The effect of fucoidan on rosetting in flow assays with parasite strain TM284R+. 100 µg/ml of fucoidan was added to TM284R+ parasite culture suspension and incubated for 30 mins before assessment of rosetting at A) 0.5 dyn/cm2 B) 0.75 dyn/cm2 and C) 1.0 dyn/cm2. The control was a culture suspension with no added fucoidan. The total rosette area (µm2) from 10 fields (20× objective) was determined after 5 minutes under flow at each shear stress using ImageJ software. Mean and standard error from three independent experiments at each shear stress are shown. No statistically significant differences were found by one-way ANOVA. The static assay data and the pooled flow data from this experiment are shown in Figure 6 of the main manuscript. (TIFF) [file pone.0073999.s003.tiff]

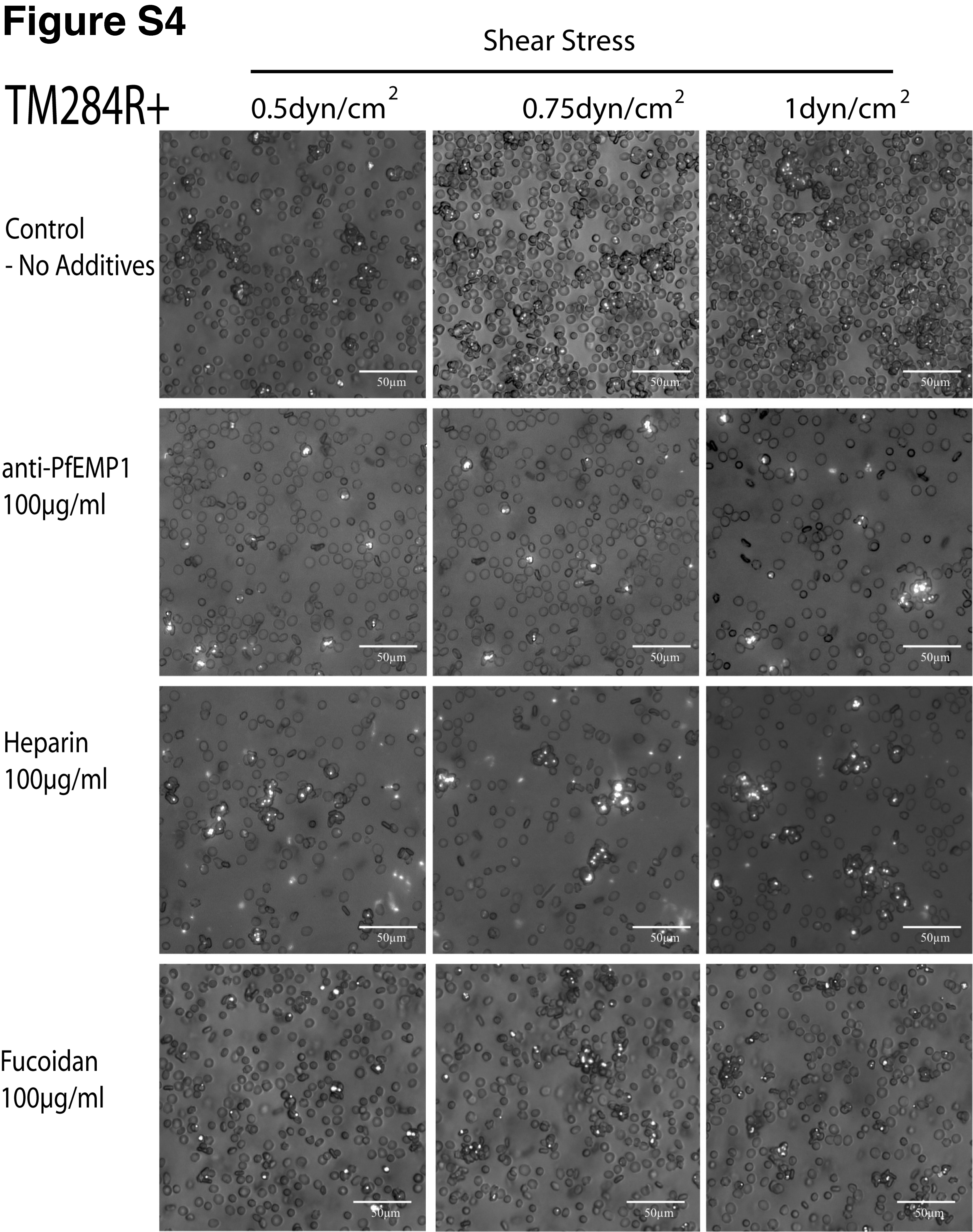

Supplement: Figure S4 — Effect of anti-rosetting agents on TM284R+ rosetting under flow. Representative images are shown for each rosetting flow experiment. (TIF) [file pone.0073999.s004.tif]
